# Solar simulated light exposure alters metabolization and genotoxicity induced by benzo[a]pyrene in human skin

**DOI:** 10.1038/s41598-018-33031-8

**Published:** 2018-10-02

**Authors:** Anne von Koschembahr, Antonia Youssef, David Béal, Clément Calissi, Etienne Bourgart, Marie Marques, Marie-Thérèse Leccia, Jean-Philippe Giot, Anne Maitre, Thierry Douki

**Affiliations:** 1 Univ. Grenoble Alpes, CEA, CNRS, INAC-SyMMES-CIBEST, Grenoble, France; 2 Equipe EPSP Environnement et Prédiction de la Santé des Populations—laboratoire TIMC (UMR CNRS 5525), CHU de Grenoble, Université Grenoble Alpes, UFR de Médecine, La Tronche, France; 3 Service de Dermatologie, allergologie et photobiologie, Centre Hospitalier Universitaire Grenoble Alpes, La Tronche, France; 4 Service de Chirurgie Plastique et Maxillo-faciale, Centre Hospitalier Universitaire Grenoble Alpes, La Tronche, France

## Abstract

Skin is a major barrier against external insults and is exposed to combinations of chemical and/or physical toxic agents. Co-exposure to the carcinogenic benzo[a]pyrene (B[a]P) and solar UV radiation is highly relevant in human health, especially in occupational safety. *In vitro* studies have suggested that UVB enhances B[a]P genotoxicity by activating the AhR pathway and overexpressing the cytochrome P450 enzymes responsible for the conversion of B[a]P into DNA damaging metabolites. Our present work involved more realistic conditions, namely *ex vivo* human skin explants and simulated sunlight (SSL) as a UV source. We found that topically applied B[a]P strongly induced expression of cutaneous cytochrome P450 genes and formation of DNA adducts. However, gene induction was significantly reduced when B[a]P was combined with SSL. Consequently, formation of BPDE-adducts was also reduced when B[a]P was associated with SSL. Similar results were obtained with primary cultures of human keratinocytes. These results indicate that UV significantly impairs B[a]P metabolism, and decreases rather than increases immediate toxicity. However, it cannot be ruled out that decreased metabolism leads to accumulation of B[a]P and delayed genotoxicity.

## Introduction

Skin is the outer barrier of the body and plays a major defensive role against chemical and physical genotoxic agents. As a consequence, the main environmental carcinogen to the skin is solar ultraviolet (UV) light^1–3^. UVB exposure is associated with direct DNA damage, namely cyclobutane pyrimidine dimers (CPD) and pyrimidine (6–4) pyrimidone photoproducts (64PP)^4^. UVA, on the other hand, is more commonly associated with oxidative stress and oxidative DNA damage^5^, but has also been shown to induce CPD^6–8^, albeit in much lower quantities compared to UVB.

Among the organic pollutants to which skin is exposed, polycyclic aromatic hydrocarbons (PAH) are ubiquitous molecules formed by incomplete combustion of organic material. Toxicity of PAH in skin is a major concern for occupational safety, where dermal contact is one of the main sources of exposure^9^. PAH as parental compounds are inert. However, metabolization via phase I enzymes, mainly cytochrome P450 (CYP) oxygenases, convert PAH into reactive metabolite intermediaries^10^. These metabolites then undergo conjugation by phase II enzymes, like glutathione-S-transferases (GST) and UDP-glucuronosyltransferases (UGT), to yield easily eliminated water-soluble compounds. Nevertheless, a fraction of phase I metabolites may diffuse to the nucleus and covalently bind to DNA forming PAH-DNA adducts^11,12^. Additionally, DNA damage may result from reactive oxygen species generated during the metabolic activation of PAH^13,14^. The association of PAH exposure and cancer risk has been observed over the years^15^, with the lungs^16^, the bladder^17^, and the skin^18^ as the main targets. While many PAH pose a significant human health risk, benzo[a]pyrene (B[a]P) has been the most studied because of its established carcinogenicity in humans^19–21^. A number of studies have focused on its highly carcinogenic metabolite, 7,8-dihydrodiol-9,10-epoxy-benzo[a]pyrene (BPDE)^22–25^. Like many other biological models, both *in vitro* and *in vivo* experiments have shown that formation of DNA adducts occurs in skin exposed to B[a]P^18,26,27^.

In regards to skin, the effects of PAH have to be discussed in terms of co-exposure with solar UV. The interaction between UV and PAH can be envisioned in different ways. First, PAH efficiently absorb UV radiation in the 290–400 nm region and can thus trigger photochemical reactions. PAH can be converted into photoproducts more toxic than their parental compounds^28,29^ or induce a photosensitized oxidative stress, as demonstrated *in vitro*^30–33^. A less studied mechanism is how UV may modulate the formation of PAH-DNA adducts. It was first shown *in vivo* that chronic co-exposure of mice to UVA and B[a]P favored the formation of BPDE adducts^34^. More recently, *in vitro* experiments have shown that UVB induced the expression of *CYP1A1* and favored the formation of DNA adducts upon subsequent exposure to B[a]P^35^.

There is still a lack of data on the combined exposure to B[a]P and UV in human skin. We designed the present study to address this point using *ex vivo* skin explants. In order to mimic real-life exposure, epidermal explants were treated topically with B[a]P and irradiations were performed with simulated sunlight (SSL) rather than pure UVA or UVB. Similar experiments were performed with primary cultures of human keratinocytes. Here, we report on how SSL modulates metabolization of B[a]P and adduct formation, whether SSL irradiations were performed before or after B[a]P treatment.

## Results

### Treatment of human skin explants and keratinocytes by B[a]P or SSL only

*Ex vivo* experiments were performed on skin explants collected from mammoplasty surgeries. Induction of metabolism was assessed by studying gene expression of Phase I and Phase II enzymes via quantitative reverse-transcription PCR (RT-qPCR). Genotoxicity was established through the quantification of BPDE-*N*^2^-dGuo, the most frequent BDPE-DNA adduct. No information was gathered on oxidative lesions, which were shown to be much less frequent than BPDE-*N*^2^-dGuo upon exposure to pure B[a]P^36,37^. After a series of experiments were performed where B[a]P was used in a dose-dependent manner (50–250 nmol) (data not shown), we chose a topical application of 100 nmol B[a]P, which was able to induce significant gene expression and BPDE-*N*^2^-dGuo formation. For SSL, we chose a dose of 2 MED, which corresponds to a relatively mild exposure likely to be encountered outdoors.

In order to determine the changes in gene expression in response to B[a]P in skin, explants from 8 different individuals were treated with 100 nmol of B[a]P over a time course of 72 hours. The shortest investigated time was 5 min, corresponding to samples exposed to B[a]P and collected as fast as possible. Expression of the phase I genes *CYP1A1*, *CYP1A*2, and *CYP1B1* was then quantified (Table 1), using untreated samples as references. Immediately after treatment, B[a]P is shown to have no effect on the relative expression of these genes. Starting at 24 hours, treatment with B[a]P significantly increases the fold change of all three CYP genes, and their expression appears to be the greatest following 48 hours of B[a]P treatment. By 72 hours, expression of these genes is significantly decreased compared to 48 hours. Relative gene expression was compared with the formation of BPDE-*N*^2^-dGuo over the same time course from the same donor samples. HPLC-MS/MS quantification of these adducts following treatment with B[a]P revealed that formation of BPDE-*N*^2^-dGuo occurs in a time-dependent manner (Table 1). Expression of the phase I enzyme EPHX and the phase II enzymes GSTA1 and GSTP1 was also studied. In general, *EPHX1*, *GSTA1* and *GSTP1* expression did not change drastically (Supplementary Table S1).

**Table 1:** Effects of pure B[a]P in human skin. For gene expression, data were collected from 8 individual donors. Samples were normalized to GAPDH used as a reference gene. Formation of BPDE-*N*^*2*^-dGuo was inferred from the analysis of 6 to 12 donors.

	Mean fold increase ± SEM			BPDE-*N*^*2*^-dGuo
	CYP1A1	CYP1A2	CYP1B1	adducts/10^6^ bases ± SEM
B[a]P 5 min	1.1 ± 0.1	1.3 ± 0.3	1.1 ± 0.2	0.0 ± 0.0
B[a]P 24 h	18.6 ± 5.3^a^	10.3 ± 3.5^a^	9.5 ± 2.1^a,d^	1.6 ± 0.4^a^
B[a]P 48 h	70.0 ± 19.4^a,b^	59.3 ± 12.3^a,c,d^	11.9 ± 1.3^a,d^	22.6 ± 9.2^a,f^
B[a]P 72 h	10.6 ± 2.0^a^	13.0 ± 7.1^e^	4.0 ± 0.2^a^	36.9 ± 11.7^c,e^

^a^Statistically different compared to 5 min (p < 0.01); ^b^Statistically different compared to 24 h and 72 h (p < 0.01); ^c^Statistically different compared to 24 h (p < 0.05); ^d^Statistically different compared to 72 h (p < 0.01); ^e^Statistically different compared to 5 min (p < 0.05); ^f^Statistically different compared to 24 h (p < 0.01).

In order to observe if SSL had any effect on relative CYP expression levels in our *ex vivo* skin explant model, we performed RT-qPCR on SSL-irradiated samples. SSL had a small effect 48 and 72 hours post-irradiation to increase expression of *CYP1A1* and *CYP1A*2, but not *CYP1B1* (Supplementary Fig. S1a). There were no relative changes for SSL irradiation on these genes at earlier time points collected. Modulation of the expression of *EPHX1*, *GSTA1* and *GSTP1* was never observed (data not shown). As expected, irradiation with SSL did not result in formation of BPDE-*N*^2^-dGuo.

Normal human keratinocytes (NHK) were cultured from the skin of the same donors as those used to prepare explants. Toxicity assays showed that a B[a]P concentration of 1 µM led to a loss of viability lower than 10%. A SSL dose of 2 MED was slightly more cytotoxic, but led only to a moderate loss of viability (less than 25% at 24 h) (Supplementary Fig. S2). These conditions were thus used in all treatments of NHK. Exposure to B[a]P led to a strong increase in the expression of CYPs, which was detectable as early as 8 hours and was much more pronounced at 24 hours (Supplementary Table S2). Like in skin, *CYP1A1* was the most over-expressed gene in HNK. However, contrary to skin, *CYP1B1* was more upregulated than *CYP1A*2. Exposure to 2 MED SSL slightly increased expression of CYP genes (fold change 2.9, 1.4 and 1.8 for *CYP1A1*, *CYP1A*2, and *CYP1B1*, respectively), but without reaching statistical significance, contrary to skin results. Increase in expression of metabolization genes upon B[a]P treatment was associated with the formation of B[a]P adducts (Supplementary Fig. S3). The level of BPDE-*N*^2^-dGuo increased in a time-dependent manner between 0 and 24 hours. Interestingly, the maximal level of adducts was not observed for the largest concentration investigated (5 µM), but rather at 1 µM. Exposure to SSL resulted in a very low overexpression of *CYP1A1* and *1B1* by a factor of 2, but not of *CYP1A*2 (Supplementary Fig. S1b). No modulation of the expression of *EPHX1*, *GSTA1* and *GSTP1* was observed (data not shown).

### SSL strongly reduces the B[a]P-mediated induction of CYP genes in skin

**Figure 1:**
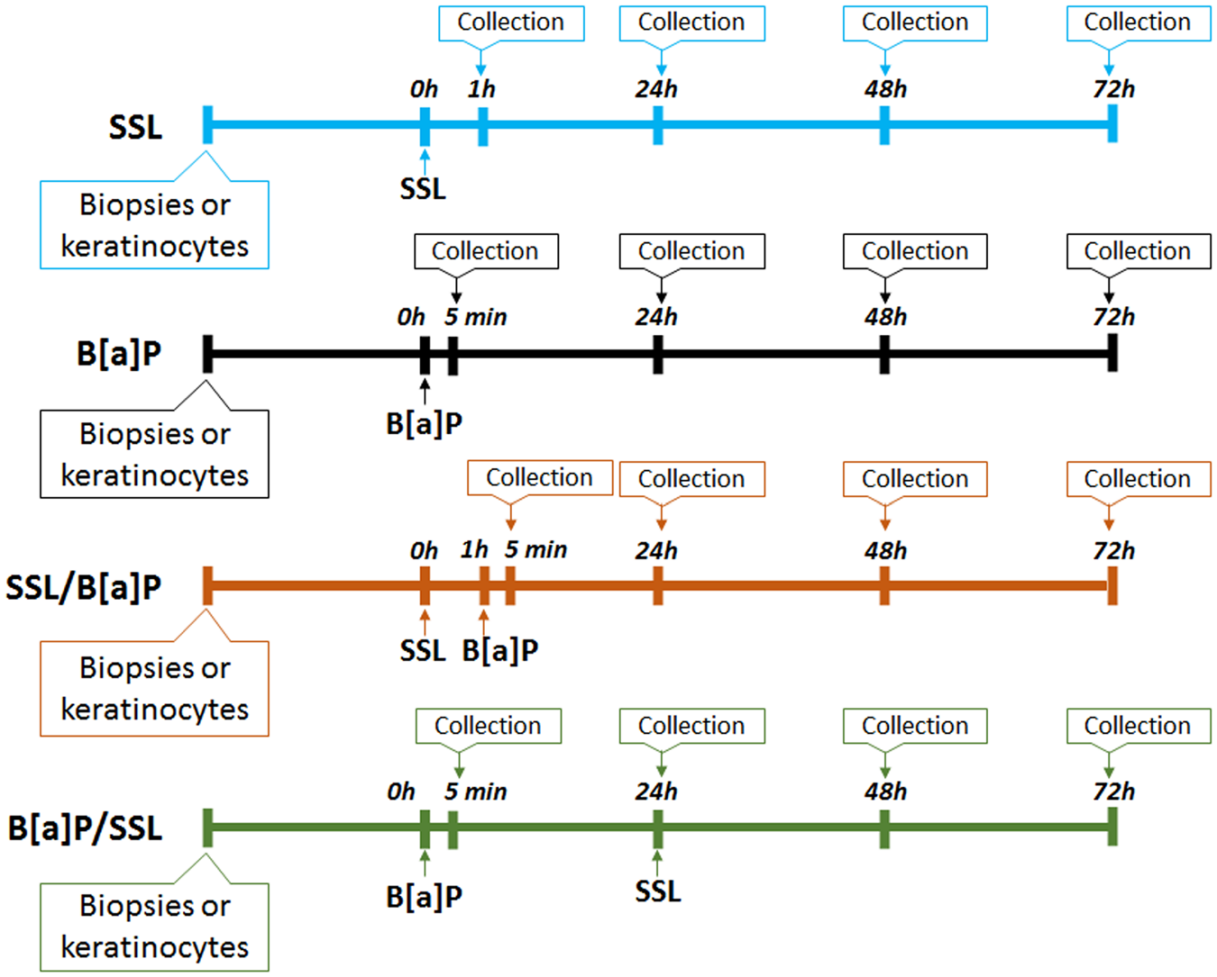
Schematic representation of experimental protocols performed. Treatment was performed topically with 100 nmol B[a]P in acetone. Samples were exposed to a dose of 2 MED of SSL. Skin explants were prepared. Three protocols were used: (i) B[a]P: explants were treated with B[a]P; (ii) SSL/B[a]P: explants were exposed to SSL and then treated 1 hour later with B[a]P and iii) B[a]P/SSL: explants were treated with B[a]P, and then exposed to SSL 24 hours later.

In order to determine what effect SSL has on cutaneous B[a]P metabolism, we compared gene expression results for explants that were treated with B[a]P versus the combination of B[a]P and SSL (Fig. 1). In addition to exposure to either SSL or B[a]P only, two treatment protocols were used: i) SSL followed 1 hour later by B[a]P exposure (SSL/B[a]P) and ii) incubation with B[a]P for 24 hours followed by UV exposure (B[a]P/SSL). The 24-hour delay after B[a]P exposure was chosen because it corresponds to the beginning of the induction of CYP genes. The time of 1 hour after SSL was selected because UV is known to induce a fast cellular response. Appropriate control samples were either exposed to vector (acetone) only, pure B[a]P or irradiated with SSL only.

**Figure 2:**
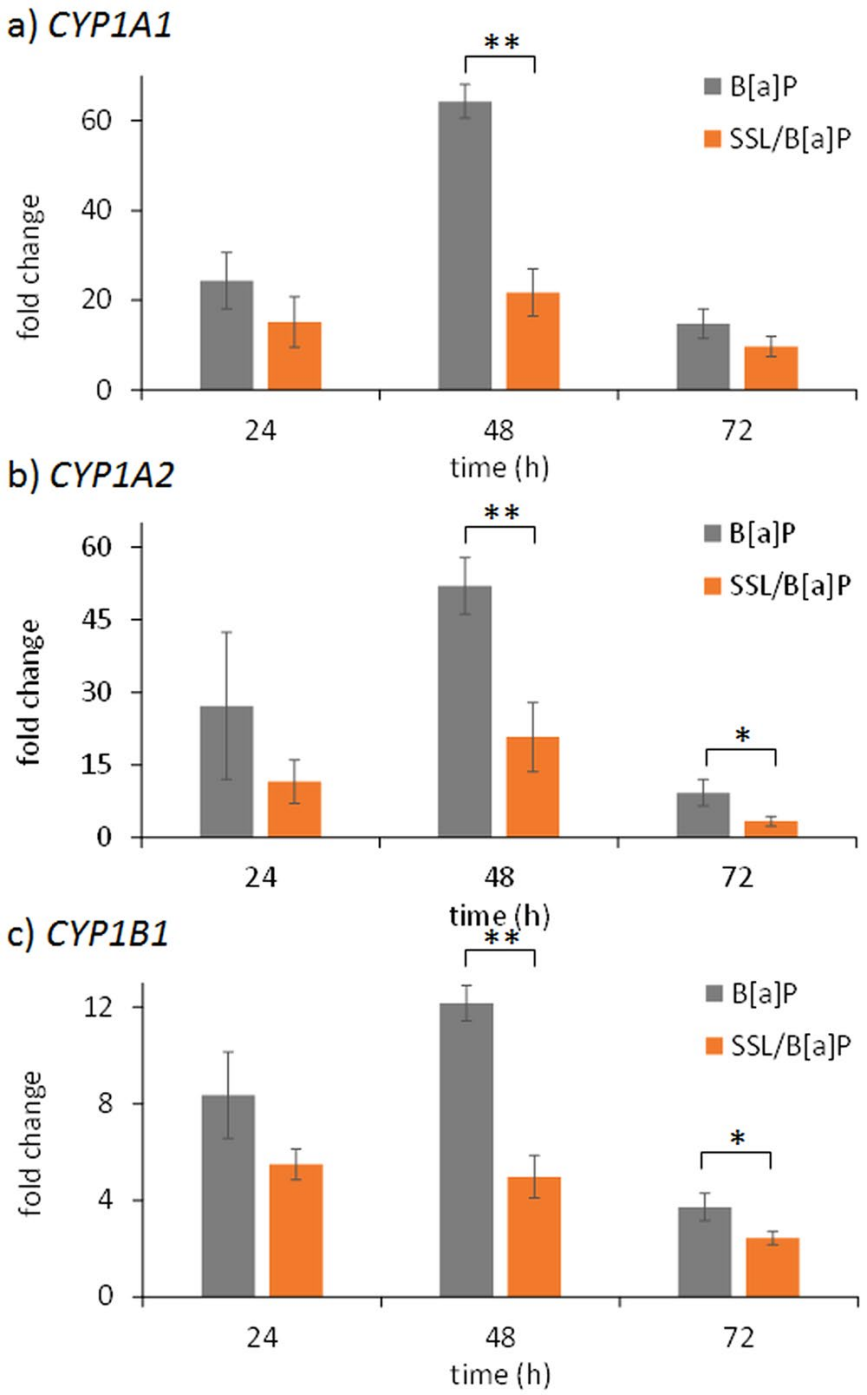
Induction of the expression of CYP genes in skin is altered when irradiation with 2 MED SSL is performed 1 hour before B[a]P treatment when compared to B[a]P treatment alone. Expression of genes was quantified at different time points by RT-qPCR at the indicated times post-treatment: (**a**) *CYP1A1*, (**b**) *CYP1A2* and (**c**) *CYP1B1*. Mean of at least three independent experiments ± SEM. Statistical significance: ^*^p < 0.05, ^**^p < 0.01.

**Figure 3:**
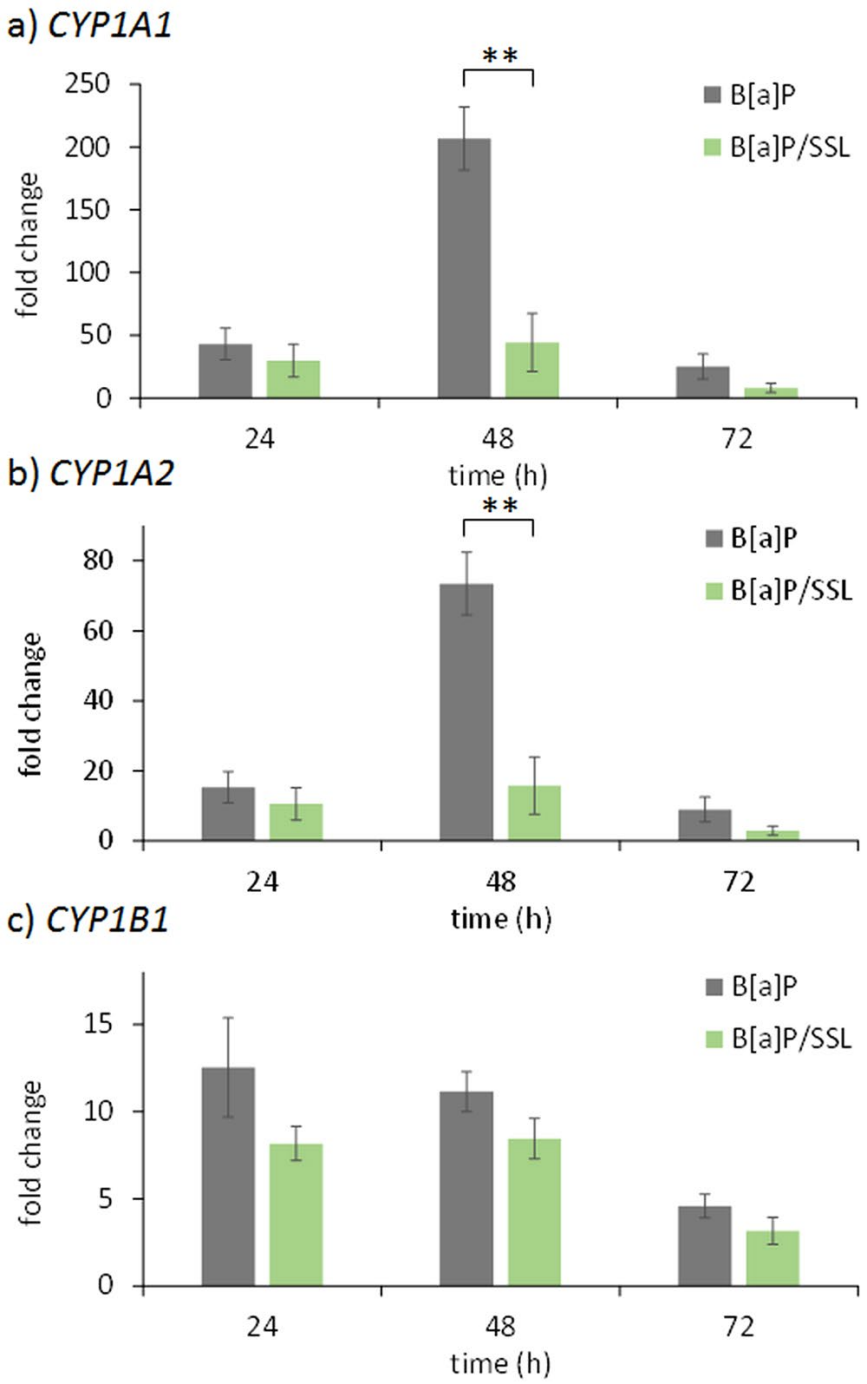
Induction of the expression of CYP genes in skin is altered when irradiation with 2 MED SSL is performed 24 hours after B[a]P treatment when compared to B[a]P treatment alone. Expression of genes was quantified at different time points by RT-qPCR at the indicated times post-treatment: (**a**) *CYP1A1*, (**b**) *CYP1A2* and (**c**) *CYP1B1*. Mean of at least three independent experiments ± SEM. Statistical significance: ^*^p < 0.05, ^**^p < 0.01.

Over the time period investigated, it was observed that SSL reduced the stimulation of gene expression induced by B[a]P of *CYP1A1* and *CYP1A*2, particularly at 48 hours and to a lesser extent at 72 hours, regardless of the treatment protocol used (Figs 2 and 3). For the SSL/B[a]P and the B[a]P/SSL protocols, the induction of these two genes represented 30 to 40% of that determined for pure B[a]P. A decreased expression of approximately 50 to 60% was still observed at 72 hours. Reduced expression of *CYP1B1* was only observed in the SSL/B[a]P protocol. We also looked to see if *EPHX1*, *GSTA1*, or *GSTP1* were altered in their mRNA expression by SSL when performed with B[a]P (Table S1). None of the combined treatments had significant effect on any of these Phase I or Phase II genes. It was only observed that *GSTP1* was slightly increased at 48 hours when human skin explants were treated by the B[a]P/SSL protocol.

### SSL reduces the formation of BPDE-*N*^2^-dGuo in human skin

**Figure 4:**
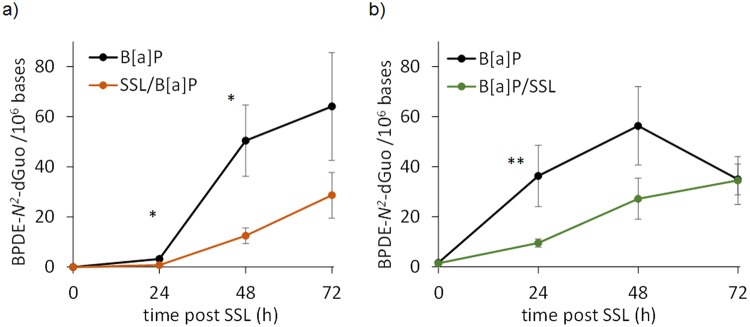
Level of BPDE-*N*^*2*^-dGuo in the DNA of skin explants treated with different protocols (**a**) SSL/B[a]P: exposure to 100 nmol B[a]P 1 hour after irradiation with 2 MED SSL and (**b**) B[a]P/SSL: exposure to 100 nmol B[a]P for 24 hours followed by irradiation with 2 MED SSL. Data were from explants from three individual donors each treated in triplicate. A comparison was made between samples exposed to B[a]P alone or to B[a]P and UV (statistical significance ^*^p < 0.05; ^**^p < 0.01).

We then quantified BPDE-adducts by HPLC-MS/MS in the DNA of human explants treated with the different protocols. BPDE-*N*^2^-dGuo was unambiguously detected on the chromatograms. Analyses clearly showed a drastic effect of co-exposure of B[a]P with SSL on the amount of adducts (Supplementary Fig. S4). In the time-course studies, irradiation with SSL appears to reduce formation of BPDE-*N*^2^-dGuo compared to B[a]P treatment alone. Under the SSL/B[a]P protocol, the amounts of BPDE-*N*^2^-dGuo are significantly lower at 24 and 48 hours post-irradiation compared to B[a]P treatment alone (Fig. 4a). The decrease at 48 hours is 5-fold. A lower level was also observed at 72 h but the difference did not reach statistical significance. We checked that the time between the end of the exposure to SSL and the addition of B[a]P, set at 1 hour in our basic protocol, did not affect the response (Supplementary Fig. S5). These data suggest a limited contribution of cell lethality on the effect of SSL on B[a]P metabolism. Indeed, the deleterious cellular processes which may ultimately lead to cell death triggered by irradiation are much more advanced 8 h than 1 h after exposure. The observation that the impact on metabolism is similar at these two time points is an indication of the limited impact of cytotoxicity on the observed reduction of formation of BPDE-DNA adducts. Under the B[a]P/SSL protocol, we observed reduced amounts of BPDE-*N*^2^-dGuo by factors of more than 2 at the 24 and 48 hour post-irradiation time points in the samples with combined treatment versus treatment only with B[a]P (Fig. 4b). By 72 hours, namely 96 h hours after the beginning of the B[a]P exposure, the samples treated with B[a]P, regardless of the combination or not with SSL, show the same amount of DNA damage (Fig. 4b), thus suggesting that SSL exposure of human skin treated with B[a]P delays formation of BPDE-*N*^2^-dGuo.

### Modulation of gene expression by SSL in B[a]P-treated NHK

**Figure 5:**
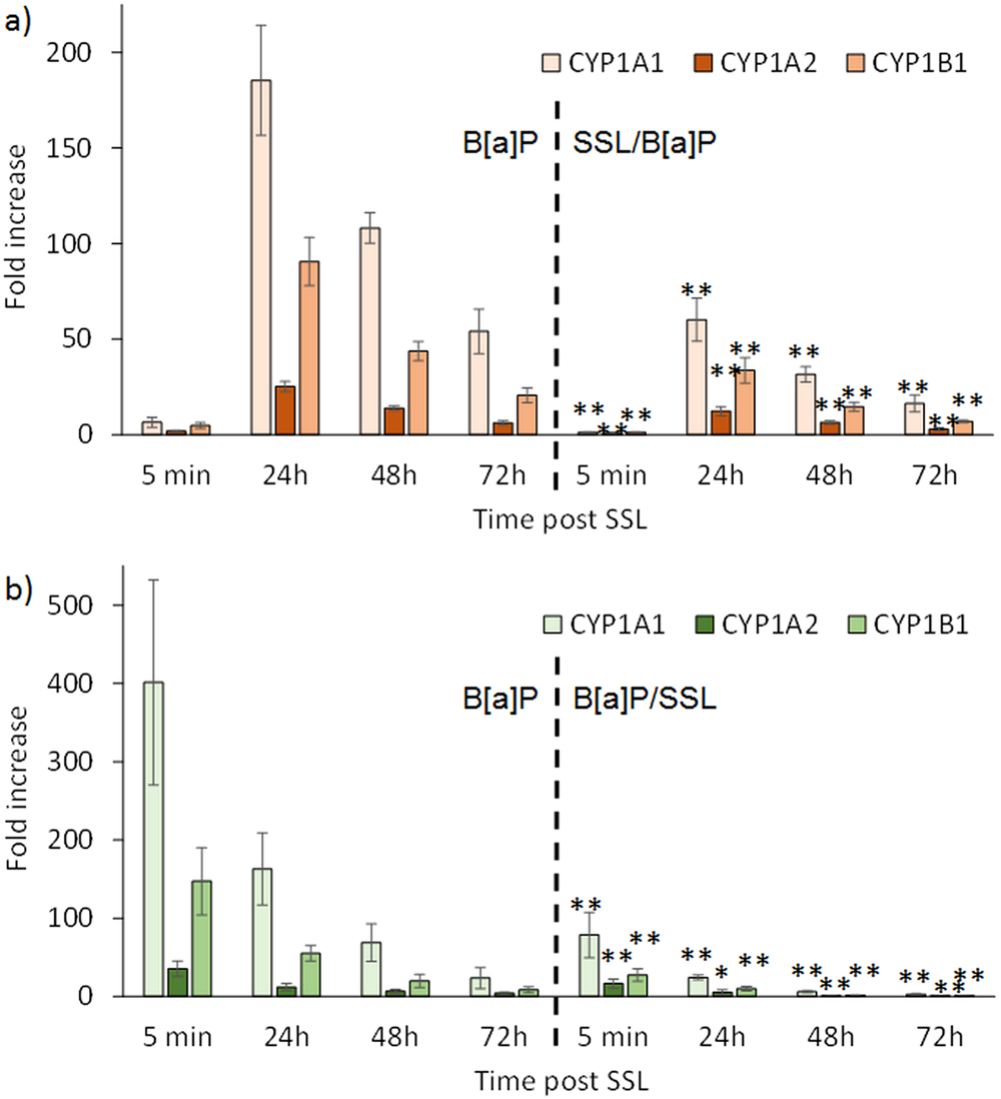
Induction of the expression of CYP genes in NHK is altered when irradiation with 2 MED SSL is performed 24 hours after B[a]P treatment when compared to B[a]P treatment alone. Expression of the genes *CYP1A1*, *CYP1A2* and *CYP1B1* was quantified at different time points by RT-qPCR in different protocols (**a**) exposure to 1 µM B[a]P 1 hour after irradiation with 2 MED SSL and (**b**) exposure to 1 µM B[a]P for 24 hours followed by irradiation with 2 MED SSL. The left side of both panel show data for unirradiated samples treated with B[a]P alone and the right side for the co-exposure protocols. Statistical significance of the differences were determined for each time point between the control and the irradiated samples. (p < 0.05, ^**^p < 0.01).

Treatment of NHK following either the SSL/B[a]P or B[a]P/SSL protocols led to a reduced induction of CYP genes than exposure to pure B[a]P (Fig. 5). This is observed for *CYP1A1*, *CYP1B1*, and *CYP1A*2 at 24, 48, and 72 hours. The ratio with respect to B[a]P only treated samples was more than 3 for *CYP1A1* and *CYP1B1* in the SSL/B[a]P protocol. Like in skin, the delay between the end of the irradiation and the addition of B[a]P did not modify the observed inhibition of BPDE-DNA adducts (Supplementary Fig. S5). The reduced gene induction effect was even larger in the B[a]P/SSL protocol were factors higher than 10 were observed at 48 and 72 hours post UV-irradiation. In contrast, no significant variation in the expression of *GSTA1*, *GSTP1* or *EPHX1* was observed (data not shown).

### Decreased formation of BPDE-*N*^2^-dGuo in NHK treated by combination of SSL and B[a]P

**Figure 6:**
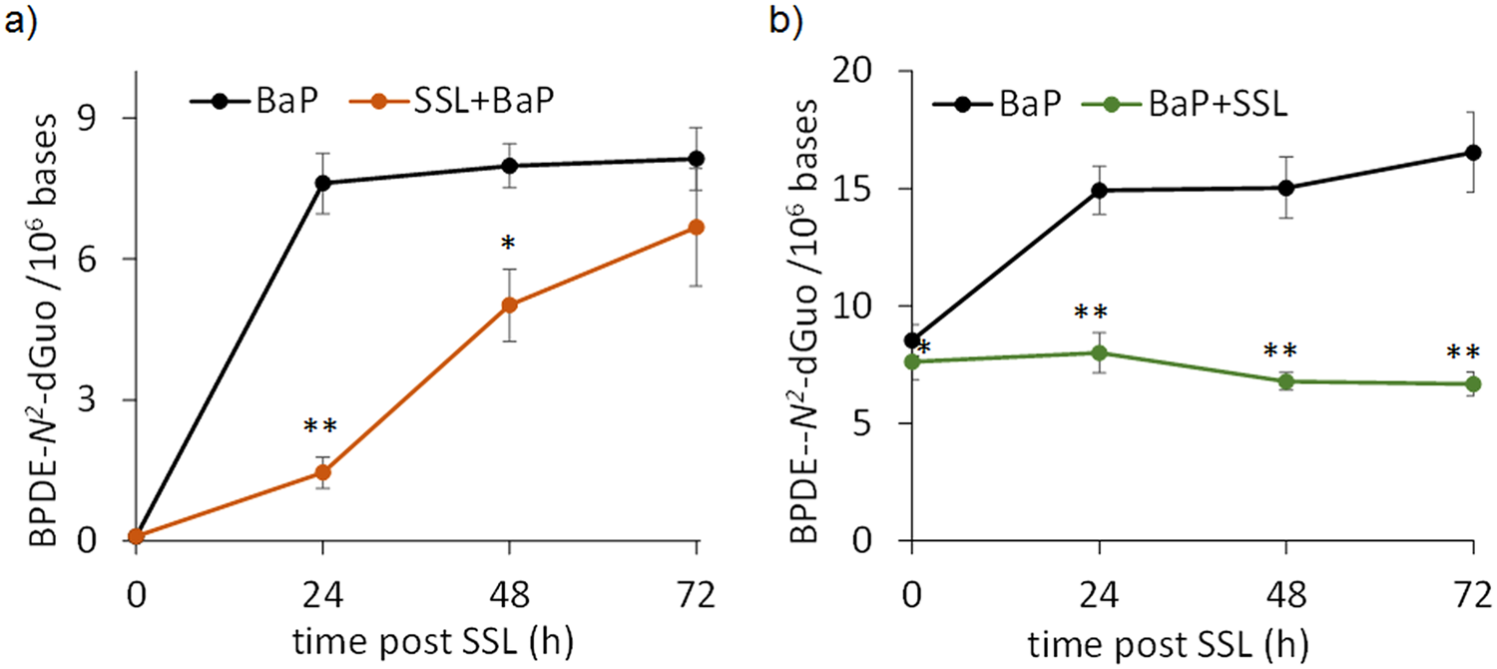
Level of BPDE-*N*^*2*^-dGuo adducts in the DNA of cultured NHK treated with different protocols (**a**) SSL/B[a]P: exposure to 1 µM B[a]P 1 hour after irradiation with 2 MED SSL and (**b**) B[a]P/SSL: exposure to 1 µM B[a]P for 24 hours followed by irradiation with 2 MED SSL. Data obtained were from cells of three individual donors each treated in triplicate. A comparison was made between samples exposed to B[a]P alone or to B[a]P and UV (statistical significance ^*^p < 0.05; ^**^p < 0.01).

SSL was also found to lead to a decrease in the yield of adducts produced in DNA upon exposure to B[a]P (Fig. 6). When exposure to SSL was performed 1 hour before addition of B[a]P, a very significant 5-fold difference on the level of BPDE-*N*^2^-dGuo was observed at 24 hours, and to a lesser extent at 48 hours (ratio 1.6). At 72 hours, the difference lost statistical significance. The impact of SSL on the formation of BPDE-*N*^2^-dGuo was even stronger when irradiation was performed 24 hours after the beginning of the B[a]P treatment. Indeed, while the level of adducts kept increasing in non-irradiated NHK, it remained constant in the cells exposed to the B[a]P/SSL protocol. The ratio between the level of adducts in cells only exposed to B[a]P and those exposed to the B[a]P/SSL treatment rose from 1.8 at 24 hours post UV to 2.4 at 72 hours.

### Effect of SSL and UVB on the formation of BPDE-dGuo adducts in HaCaT cells

We treated HaCaT cells under conditions which were not more cytotoxic than in NHK (Supplementary Table 3). BPDE-*N*^2^-dGuo was found to be produced in an almost 10-times larger yield than in primary keratinocytes. Exposure of cells to SSL either 1 hour before or 24 hours after treatment with B[a]P resulted in a significant decrease in the formation of BPDE-*N*^2^-dGuo in DNA (Supplementary Fig. S6a). Exposure to UVB at a dose of 6 mJ/cm^2^ resulted in a decrease in the level of adduct at 48 hours when irradiation was performed before B[a]P treatment (Supplementary Fig. S6b). This UVB dose did not modulate the level of adducts at earlier times points or when exposure to UVB occurred after B[a]P treatment. The higher dose of UVB (20 mJ/cm^2^) led to a lesser decrease in the level of BPDE-*N*^2^-dGuo (Supplementary Fig. S6c).

## Discussion

The combined effects of UV and PAH in skin has major implications in human health, particularly in occupational safety. Indeed, cutaneous exposure is a much more important contamination source in workers than in the general population. As a consequence, their skin is more likely to experience exposure to solar UV in the presence of PAH. In order to provide valuable data on this subject, we performed a series of *ex vivo* and *in vitro* experiments aimed at determining whether exposure to solar UV radiation modulates the metabolization of B[a]P and the formation of BPDE-*N*^2^-dGuo after B[a]P treatment.

The first part of our study involved explants made from freshly collected human skin. While there have been numerous studies of B[a]P metabolism performed in animal models, human skin has been less used as a working model. Costa *et al*.^38^ used punch biopsies of abdominal skin to test the effect of B[a]P on expression of *CYP1A1*. Brinkmann *et al*.^39^ also used human skin to test out genotoxic effects of B[a]P, particularly on metabolite formation and DNA damage, as determined by the comet assay. In order to provide further insights of the effects of B[a]P in skin, we first quantified the expression of various genes involved in xenobiotic metabolism. Emphasis was placed on classical metabolic enzymes *CYP1A1*, *CYP1B1*, and *CYP1A*2 found in the skin and other organs^40,41^. We also included other genes expressed in skin, such as *GST* and *EPHX*^39,42,43^. Expression of these same genes was also quantified in human keratinocytes grown from the skin of the same donors.

In agreement with Costa *et al*.^38^, our RT-qPCR analyses unambiguously showed that exposure of human skin and NHK^41,44^ to B[a]P results in a time-dependent increase of *CYP1A1*, *CYP1A*2, and *CYP1B1*. A lag period of less than 24 hours and a maximal expression at 48 hours were observed in skin. Induction of metabolism genes was faster in NHK with a lag phase of 8 hours and a maximal expression at 24 hours. Interestingly, induction of *CYP1A*2 in skin was found to be much larger than that of *CYP1B1*, whereas an opposite trend was found in NHK, similar to other cell types^36^. No significant modulation of the expression of phase II enzymes was observed. The temporal pattern observed for the formation of BPDE-*N*^2^-dGuo in skin also included a lag phase of approximately 24 hours and then reached a maximum at 72 hours. The shape of the time-course of formation of BPDE-*N*^2^-dGuo formation exhibiting a lag phase is reminiscent of data obtained in numerous cell types^36,37,45,46^. It corresponds to an induction period of phase I metabolization enzymes clearly observed on the time-course for gene expression which is maximal after 48 hours. Consequently, the production of metabolites with DNA-damaging capacities does not take place immediately after the beginning of the treatment. It may be added that the level of adducts tends to decrease after 72 hours. This likely reflects the repair of BPDE-*N*^2^-dGuo via nucleotide excision repair, which is quite slow^47,48^. Altogether, these data validate that *ex vivo* human skin explants are suitable model for studying PAH metabolization and genotoxicity under our experimental conditions.

We then investigated the impact of exposure to SSL on B[a]P-mediated stimulation of metabolization and genotoxicity of B[a]P, which has yet to be studied. Based on available literature, we anticipated significant synergistic effects. Experiments in mice involving chronic exposure to B[a]P and UVA showed synergistic effects on the formation of B[a]P adducts^34^, possibly due to decreased repair capacities. Moreover, UVB was found to increase expression of *CYP1B1* in human keratinocytes^44^ and HaCaT cells^49^, and it was suggested that this occurred through a signal transduction pathway involving photoactivated tryptophan and its oxidation photoproduct 6-formylindolo[3,2-b]carbazole (FICZ), a known ligand of the AhR^49,50^. Katiyar *et al*.^42^ reported that CYP1A1 and CYP1B1 were induced following UV exposure at both the gene and protein level in human epidermis. Finally, Nair *et al*.^35^ observed that UVB exposure of HaCaT cells favored the formation of BPDE adducts following B[a]P treatment. Accordingly, we report that UV alone increases expression of *CYP1A1* by a factor approximately 5 in skin and 2 in NHK, in the same range of low values observed by others^35,42,49,51^. It should be stressed that this stimulation of the expression of CYP genes remains modest compared to the almost two orders of magnitude larger expression following B[a]P treatment.

We did not observe the same modest stimulation of CYP expression by SSL in the combination with exposure to B[a]P either before or after irradiation. Our experiments involving irradiation followed by B[a]P treatment rather showed that preliminary irradiation of skin explants strongly inhibited the induction of Phase I *CYP1A1*, *CYP1A*2, or *CYP1B1* metabolism genes. Reduced expression of metabolic genes is associated with a lower level of BPDE-*N*^*2*^-dGuo in skin exposed to both SSL and B[a]P compared to those exposed to B[a]P only. This result unambiguously shows that UV decreased the metabolite load in the skin exposed to the SSL/B[a]P protocol. The same was true for the converse B[a]P/SSL protocol. Therefore, we can conclude that SSL has a repressive effect on B[a]P-induced metabolism in our *ex vivo* skin model. The response of skin explants seems thus to be drastically different from that of HaCaT cells, where combined UVB/B[a]P treatment was reported to favor formation of BPDE-DNA adducts^35^.

The differences between our observations in explants and published *in vitro* data could have been explained by the fact that most keratinocytes in skin are differentiated and may exhibit different metabolizing properties from cultured cells. This possibility led us to investigate the effect of a combined exposure to SSL and B[a]P in cultured, undifferentiated keratinocytes taken from the same skin samples. We made the same observations of a decreased expression of CYP genes and formation of BPDE-*N*^*2*^-dGuo in this model as in skin. This was expected since keratinocyte is the main cell type responsible for metabolism of xenobiotics in skin. Our results also show that differentiation and cutaneous environment do not explain the differences between our results and the published *in vitro* increase in B[a]P-DNA adduct in UV-exposed cells^35^.

The main difference between our work and that reporting the favored formation B[a]P-DNA adducts in UV-exposed cells is the cellular model. While we used human skin explants and primary cultures of human keratinocytes, other works involved the keratinocyte cell line HaCaT^35^ known to be mutated, particularly in the P53 gene. We also observed that HaCaT are more metabolically active than NHK since they induce an almost 10-time larger amount of BPDE-*N*^*2*^-dGuo. In order to determine whether the discrepancies were explained by the difference in models, we applied the SSL/B[a]P and B[a]P/SSL protocols to HaCaT cells. A strong inhibition in the formation of adducts was observed when compared to treatment with pure B[a]P, like in skin and NHK. Another difference between our work and previously published data was the nature of the radiation applied to the samples. Instead of the more biologically relevant SSL, the authors used UVB. We thus repeated the co-treatment protocols on HaCaT with UVB instead of SSL. A low dose of 6 mJ/cm^2^, similar to that used by Nair *et al*.^35^, was applied and we also observed a decrease, rather than an increase, in the level of B[a]P adducts when irradiation was performed before B[a]P treatment. A larger UVB dose (20 mJ/cm^2^) was also used, which resulted also in a decreased formation of BPDE-*N*^*2*^-dGuo upon B[a]P treatment, although to a lesser extent than with SSL or 6 mJ/cm^2^ UVB. These observations show that UVB is less efficient than UVA at inhibiting the formation of DNA adducts, at least in HaCat cells. However, this does not explain why increased formation of adducts was reported with UVB in HaCaT cells^35^. One possible explanation may be the difference in the assays used for the quantification of DNA adducts. While we directly targeted them in enzymatically hydrolyzed DNA, the previous work relied on an indirect measurement based on HPLC-fluorescence detection of B[a]P-tetraol released by acidic hydrolysis of extracted DNA. Another difference is that irradiations in the previous work were performed in phenol-free DMEM medium while we used PBS in order to prevent any photosensitization processes. Altogether, we could confirm in HaCaT cells the inhibitory effects of UV on the metabolism and genotoxicity of B[a]P that we observed in skin and NHK.

Our results on skin and NHK show that when a UV source mimicking sunlight is used, no increased metabolization and genotoxicity of added B[a]P takes place. Yet, as observed by others^35,42,49,51^, SSL alone lead to a small over expression of CYP genes. These results suggest that the endogenous photoproducts mediating the activation of B[a]P metabolism are either weak ligands of AhR or produced in low amounts since exogenous ligands efficiently compete for the binding to AhR. Consequently, the role of FICZ in the effects of combined exposures to UV and B[a]P or other PAH may be smaller than anticipated^35,50,51^. Actually, the presence of this compound in skin has never been shown. Formation of FICZ has only be detected *in vitro* in HaCaT cells under non-physiological conditions^49^. In addition, it should be reminded that tryptophan is a rare amino acid amongst the less frequent ones in proteins^52^. We recently published that FICZ is a minor photoproduct of tryptophan^53^ partly because its formation requires addition of two tryptophan photoproducts^54^ and its yield depends quadratically on the dose. The fact that SSL irradiation exhibits a strong inhibitory effect when applied 24 hours after BaP treatment (B[a]P/SSL protocol), namely when the AhR pathway has been extensively triggered, reinforces the idea of a limited role of the FICZ in the modulation of B[a]P metabolism in skin.

Alternative explanations have thus to be found to our observations. Obviously, FICZ-mediated activation of AhR cannot account for our results. Photoproducts arising from various cellular components may yet play a role in the inhibition the metabolization of B[a]P, but rather as competitor with CYPs, as observed in mixtures of PAH^55–58^. However, the latter process does not explain the massive reduction in expression of CYP genes. More general cellular stresses may also be involved, rather than a cellular response involving specific pathways. First, the reduced expression of CYP genes could be explained by the well-documented temporary arrest in RNA synthesis in transcribed genes that takes place following UV irradiation^59–61^. Another explanation could be related to the ability of UV to deplete the cellular nicotinamide adenine dinucleotide (NADH) pool^62^. This important biological factor is key in the production of ATP and in the cellular energy content^63^. It seems likely that reduced energy level leads to decrease metabolization capacities. NADH is also a major cofactor as a reductant in the enzymatic mechanism of CYP450^64^. Therefore, decreased intracellular NADH concentration could explain the decreased metabolization of B[a]P. The observation of similar effects when SSL is applied either before or after B[a]P treatment also rules out a major contribution of photodegradation of the PAH since B[a]P is never irradiated in the SSL/B[a]P protocol.

In conclusion, our study provides for the first time an extensive picture of the impact of SSL, namely a combination of both UVB and UVA components, on the metabolism and genotoxicity of B[a]P. In contrast to previous data obtained *in vitro* with pure UVB, solar UV radiation appears to have a strong repressive effect on B[a]P-induced gene induction of CYP enzymes and delays formation of BPDE-DNA adducts in skin. Although positive in terms of immediate impact, this result may also suggest that parent PAH accumulate longer in irradiated skin. Indeed, only metabolites are actively eliminated from the epidermis^65^. Therefore, it is possible that UV exposure converts an acute exposure to a high dose into a more chronic exposure to a lower dose. It is unclear what impact this would have on influencing or initiating skin cancer. Another possible mechanism to increased genotoxicity upon co-exposure to PAH and UV is an impact on the formation and repair of bipyrimidine photoproducts. We are currently working on this topic by using an approach similar to that used in the present work.

## Methods

### Skin explants preparation

Human skin samples were obtained following breast surgery from heathy female donors with their informed consent (Centre Hospitalier Universitaire de Grenoble, Grenoble, France). All experiments were performed in accordance with relevant guidelines and regulations. In particular, work was performed in agreement with article L1245-2 of the French Public Health Code on the use of surgical wastes for research purposes. It stipulates that donors must be informed and agree; and that samples are used anonymously. Collection and storage of human skin samples was declared to the French authorities and validated in the CODECOH DC-2008-444 document. All donors were between the ages of 17–58, and their skin phototype was between I and III, according to the Fitzpatrick classification scale^66^. Skin samples were handled as previously described^7^. Epidermal skin was dermatomized to a 0.25 mm thickness using a SOBER hand-held dermatome (Humeca, Enschede, Netherlands), and then 12-mm punch biopsies were made. Samples were placed in 12-well plates and maintained via liquid/air interface in DMEM/F-12 medium (Thermo-Fisher) containing 10% Pen/Strep at 37 °C in a humidified incubator containing 5% CO_2_.

### UV irradiation of human skin explants

SSL irradiations were performed using a LS1000 Solar Simulator (Solar Light Company, Glenside, PA), which emitted wavelengths in the 290–400 nm range. Live radiation intensity was measured using the Erythema Meter, and displayed as live intensity (minimal erythema dose (MED)/hr). In all experiments, the dose of 2 MED was used for all irradiated samples. Prior to irradiation, culture media was removed and the samples were rinsed twice with PBS. Irradiations were performed at room temperature in PBS via liquid/air interface without the plate cover. Following irradiations, PBS was replaced with fresh culture medium, and the samples were kept in the incubator until their collection time, when explants were halved. One series of samples was not replaced in the incubator but frozen, corresponding to a post-irradiation time of 5 min. Samples for DNA damage quantification were immediately frozen at −20 °C whereas samples used for gene expression analysis were stored at −80 °C in RNAlater (Thermo Fisher).

### Treatment of skin

Different treatment protocols were applied to the skin explants: i) Exposure to SSL only, II) exposure to B [a]P only, iii) SSL exposure followed 1 hour later by B[a]P treatment (SSL/B[a]P), and iv) incubation with B[a]P for 24 hours followed by exposure to SSL (B[a]P/SSL). For samples treated under the SSL/B[a]P protocol, SSL irradiations were performed immediately following biopsy preparation. After irradiation, samples were maintained in PBS in the incubator for 1 hour. PBS was removed, and samples were treated with vector (100% acetone, Sigma-Aldrich, Lyon, France) or 5 µL of a 20 mM B[a]P (Sigma-Aldrich) solution. Fresh culture medium was added and samples placed in the incubator. They were collected after increasing periods of time. One series of samples was not placed in the incubator but frozen immediately, corresponding to a post-irradiation time of 5 minutes. For samples treated under the B[a]P/SSL protocol, pre-treatment with vector (100% acetone) or 5 µL of 20 mM B[a]P was performed immediately following biopsy preparation. Fresh culture media was added to each well and the samples were maintained in the incubator. Twenty-four hours later, culture medium was removed, and the biopsies were gently washed twice in PBS. SSL irradiations and samples collection was performed after increasing periods of time. One series of samples was not placed in the incubator but frozen immediately, corresponding to a post-irradiation time of 5 minutes. For all treatment protocols, the minimum number of human donors was 3, with samples collected in triplicates.

### Treatment of cultured keratinocytes

Keratinocytes were obtained from fresh human skin samples, as previously described^7^. Cells were grown in KSF medium containing 1% Pen/Strep, 25 µg/mL bovine pituitary extract (BPE), and 0.9 ng/mL of recombinant human epidermal growth factor (EGF). BPE and EGF were removed from the cell culture media 24 hours before treatment or irradiation, and throughout the experiment performed. For samples treated under the SSL/B[a]P protocol, keratinocytes were rinsed twice in PBS and exposed to 2 MED SSL in PBS. They were then maintained in PBS in the incubator for 1 hour. B[a]P dissolved in DMSO was then added to reach a final concentration of 1 µM (Sigma-Aldrich). Samples were collected after increasing periods of time. Untreated controls were incubated with pure DMSO. For samples treated under the B[a]P/SSL protocol, pre-treatment with vector (DMSO at a final concentration of 1%) or 1 µM B[a]P was performed in the incubator. Twenty-four hours later, culture medium was removed and the cells were washed twice in PBS. SSL irradiations and samples collection was performed, as described above. For both protocols, one series of samples was not placed in the incubator after irradiation but frozen immediately, corresponding to a post-irradiation time of 5 min.

### Quantitative reverse transcription PCR

Gene expression analysis was performed on phase I (*CYP1A1*, *CYP1A2*, *CYP 1B1*, and *EPHX1*) and phase II (*GSTA1* and *GSTP1*) metabolism genes. Total RNA was extracted following treatment by homogenizing the skin with the TissueRuptor, Proteinase K digestion, QiaShredder column, and RNeasy Mini Kit combined with the RNase-free DNase system (Qiagen, Courtaboeuf, France). RNA samples were quantified using a ND1000 NanoDrop spectrophotometer. First strand cDNA was synthesized from 1.0 µg of total RNA using the SuperScript VILO Synthesis Kit (Thermo-Fisher), and quantitative real-time polymerase chain reaction (RT-qPCR) was performed using the appropriate human RT^2^ qPCR primers and RT² SYBR Green qPCR Mastermix (Qiagen). Amplification was performed using the Bio-Rad CFX96 system (Bio-Rad, Marnes-la-Coquette, France). The cycle threshold (CT) values were determined using the Bio-Rad CFX96 system software, and the comparative ΔΔCT method was used to calculate the relative fold change of all genes after normalization to *GAPDH*, *RPS18*, or *β-Actin* (Livak and Schmittgen, 2001). Biological triplicates were processed individually for RNA isolation and analyzed in technical duplicates. Values were normalized to expression in control (untreated) samples.

### Quantification of BPDE-*N*^*2*^-dGuo

For DNA extraction, samples were homogenized using the TissueLyzer II and extracted using the DNeasy Blood and Tissue Kit, both from Qiagen. Explants or cell pellets were homogenized using the ATL lysis buffer combined with mechanical disruption using metal beads, followed by treatment with proteinase K. Samples were then treated with RNase-A before a second lysis step using AL buffer, before loading onto DNeasy spin columns. DNA was eluted off the column by adding 200 µL of water in two separate, consecutive centrifugations. Samples are then freeze-dried overnight at −20 °C and then rehydrated the next day in 50 µL of water. Hydrolysis of DNA samples was performed using a cocktail of enzymes. The first incubation was performed using phosphodiesterase II, DNase II, and nuclease P1 (37 °C, 30 minutes). The second incubation step was then performed using phosphodiesterase I and alkaline phosphatase (37 °C, 2 hours). All enzymes used were purchased from Sigma-Aldrich.

Samples were then injected on an HPLC system (Agilent, Massy, France) connected to a reverse-phase HPLC column (150 × 2 mm ID, 3 μm particle size, ODB, Interchim, Montluçon, France). The mobile phase was a gradient of acetonitrile (6 to 80%) in 2 mM ammonium formate (pH 6). BPDE-*N*^*2*^-dGuo was detected by a tandem mass spectrometer (SCIX API 3000) using transitions m/z 570→257 and m/z 570→454 as previously described^36,37,67^. Unmodified nucleosides were quantified with a UV detector. External calibration of the response of the detector was performed for each run of analyses. Repeated injections of standards during the run permitted to control the stability of the sensitivity of the detection and of the retention times. Results were expressed in number of adducts per million normal bases.

### Culture and treatment of HaCaT cells

HaCaT cells were grown in DMEM medium in the presence of Pen/Strep and FBS (10% v/v). For treatment, cells were grown in 6 cm diameter petri dishes. Prior to irradiation, the medium was removed and replaced by PBS. Exposure to SSL was performed by using a LS1000 Solar Simulator (Solar Light Company, Glenside, PA) with a dose rate of 6 MED/h. UVB irradiations were performed with a 2 × 15 W UVB lamp exhibiting an emission spectrum centered at 312 nm. The dose rate was 70 mJ/cm^2^ per min. For the treatment with B[a]P, cells were placed in culture medium without FBS. A stock solution of B[a]P in DMSO was added to reach the selected concentration. In all experiments, cells were collected after 24 and 48 hours post-treatment. MTT tests and quantification of BPDE-*N*^*2*^-dGuo were performed, as previously described for keratinocytes. Similar protocols were used for the combination experiments with SSL set at 2 MED, UVB used at either 6 or 20 mJ/cm^2^ and B[a]P at 1 µM.

### Statistical analysis

Statistical analysis were performed on pools of replicates originating from different donors. Data were statistically analyzed in GraphPad PRISM using paired t-tests with non-parametric Wilcoxon, or one-way ANOVA followed by Student-Newman-Keuls (SNK) for multiple comparisons.

## Electronic supplementary material

Supplementary Information
