# Supplementary material for: Solar simulated light exposure alters metabolization and genotoxicity induced by benzo[a]pyrene in human skin
Source: Sci Rep. 2018 Oct 2;8:14692. doi: 10.1038/s41598-018-33031-8 (PMC6168490; doi:10.1038/s41598-018-33031-8)

Solar simulated light exposure alters metabolization and genotoxicity induced by benzo[a]pyrene in human skin

Anne von Koschembahr, Antonia Youssef, David Béal, Clément Calissi, Etienne Bourgart, Marie Marques, Marie-Thérèse Leccia, Jean-Philippe Giot, Anne Maitre and Thierry Douki

Supplementary Information

1. Table S1: Expression of some Phase I and phase II enzymes in B[a]P-treated skin

Table S1 shows the limited effect of B[a]P alone or in combination with SSL on the expression of *EPHX1*, *GSTA1* and *GSTP1*. Data were collected from 3 or 4 individual donors. Samples were normalized to *GAPDH* used as a reference gene. Results are mean fold change ± SEM.

**SSL/B[a]P**						
	24 h		48 h		72 h	
	B[a]P	SSL+B[a]P	B[a]P	SSL+B[a]P	B[a]P	SSL+B[a]P
EPHX1	1.5 ± 0.6	0.8 ± 0.2	3.0 ± 2.1	0.8 ± 0.4	0.5 ± 0.0	0.2 ± 0.0
GST A1	2.3 ± 1.2	1.3 ± 0.5	2.2 ± 1.4	1.3 ± 0.8	1.1 ± 0.5	1.8 ± 0.9
GST P1	1.1 ± 0.1	1.1 ± 0.1	1.2 ± 0.1	0.8 ± 0.1	1.0 ± 0.3	1.0 ± 0.0
**B[a]P/SSL**						
	24 h		48 h		72 h	
	B[a]P	SSL+B[a]P	B[a]P	SSL+B[a]P	B[a]P	SSL+B[a]P
EPHX1	1.2 ± 0.1	1.4 ± 0.4	1.4 ± 0.4	1.2 ± 0.5	0.8 ± 0.0	0.1 ± 0.0
GSTA1	3.1 ± 1.4	0.8 ± 0.4	2.8 ± 1.3	1.5 ± 0.5	1.1 ± 0.4	1.2 ± 0.8
GSTP1	1.0 ± 0.1	1.1 ± 0.1	0.9 ± 0.1	1.5 ± 0.2 [1]	0.8 ± 0.2	0.8 ± 0.1

[1]Statistically different from B[a]P 48h and from B[a]P+SSL 24h ($p<0.05$)

2. Figure S1. Induction of CYP genes upon exposure of pure SSL

Skin explants (a) and NHK samples (b) were exposed to SSL in PBS and replaced in fresh medium. They were placed in the incubator for increasing period of times. Samples were then collected and expression of CYP genes was quantified by RT-qPCR. Reported values are means ± SEM (n=6).

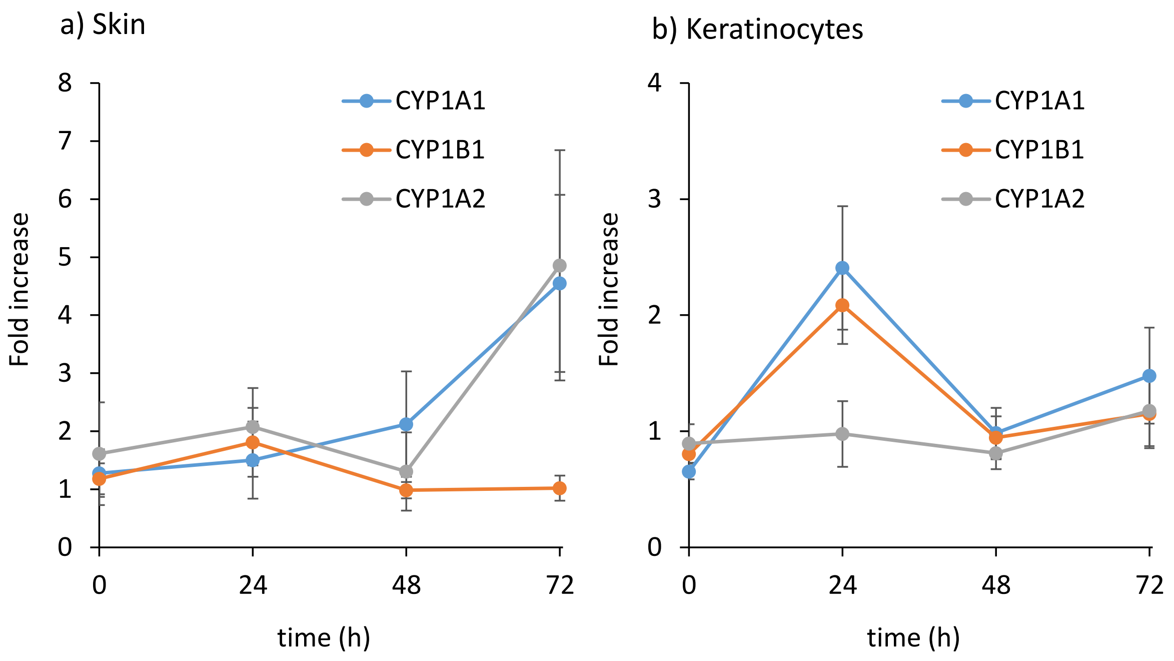

3. Figure S2. Toxicity of B[a]P and SSL in NHK

Dose- and time-dependent loss of viability in cultured NHK exposed to either a) SSL or b) B[a]P. panel c) is the extension for the low concentrations of the B[a]P data. Cellular viability was evaluated at 24, 48, or 72 hours using the MTT assay. Cells obtained from 6 individual donors were used and studied in triplicate. Results are % of viability with respect to untreated cells ± SEM.

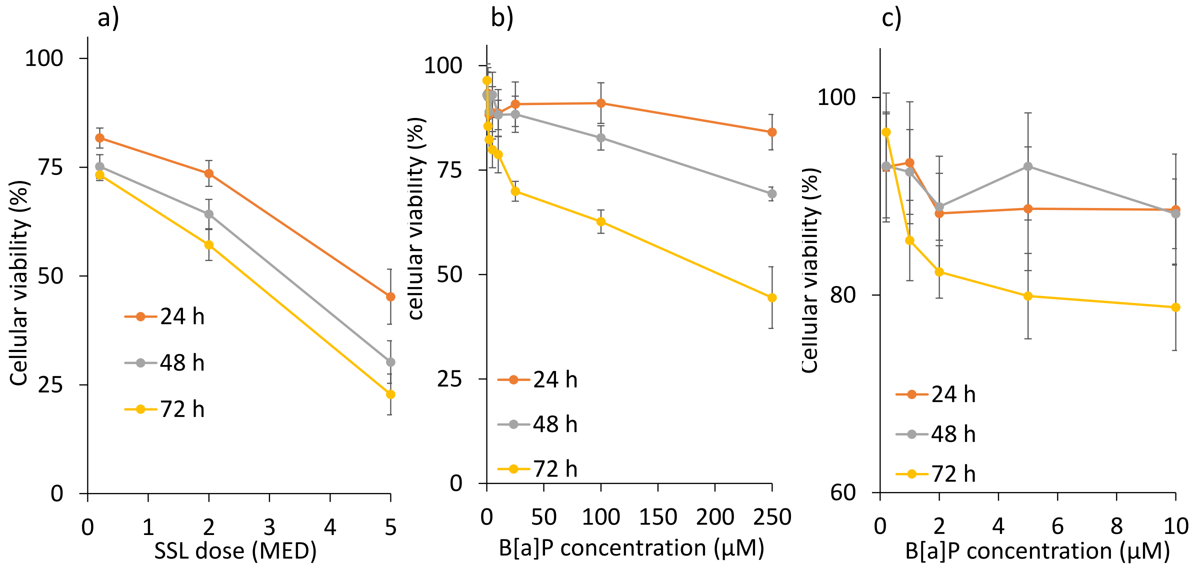

4. Table S2: Expression of some Phase I and phase II enzymes in B[a]P-treated NHK

Table S2 shows the effect of exposure of NHK to pure B[a]P on the expression of several genes. While CYP450 are efficiently overexpressed, other genes coding for other metabolism enzymes are not modulated *(EPHX1*, *GSTA1* and *GSTP1*). Factors involved in induction of PAH metabolism (*AhR* and *ARNT*) do not appear to be modulated either. Data were collected from primary NHK cultures from 3 individual donors all studied in triplicates. Samples were normalized to *GAPDH* used as a reference gene. Results are mean fold change ± SEM.

Time	2h		8h		24h	
B[a]P	0.2 µM	2 µM	0.2 µM	2 µM	0.2 µM	2 µM
CYP1A1	10 ± 1	12 ± 1	51 ± 33	80 ± 64	155 ± 86	948 ± 317
CYP1A2	4 ± 1	4 ± 2	8 ± 4	12 ± 7	17 ± 4	57 ± 22
CYP1B1	8 ± 2	10 ± 2	42 ± 18	55 ± 35	97 ± 64	497 ± 227
GSTA1	1.5 ± 0.4	1.4 ± 0.4	0.4 ± 0.1	0.5 ± 0.2	0.9 ± 0.6	0.6 ± 0.1
GSTP1	0.9 ± 0.1	0.9 ± 0.1	0.9 ± 0.1	1.0 ± 0.2	1.2 ± 0.1	1.4 ± 0.1
EPHX1	1.1 ± 0.1	1.1 ± 0.1	1.0 ± 0.1	1.0 ± 0.0	0.9 ± 0.0	1.0 ± 0.1
AhR	0.9 ± 0.1	0.8 ± 0.1	0.8 ± 0.1	0.8 ± 0.2	0.7 ± 0.1	0.6 ± 0.3
ARNT	1.3 ± 0.3	1.3 ± 0.2	0.9 ± 0.1	0.9 ± 0.1	1.0 ± 0.1	1.0 ± 0.1

5. Figure S3. Formation of BPDE-N^2-dGuo in B[a]P-treated NHK

NHK were cultured from skin biopsies. They were then exposed to B[a]P dissolved in DMSO added to the culture medium. The final concentration ranged between 0.2 and 5 µM. After different periods of time, cells were collected, DNA extracted and the level of BPDE-N^2-dGuo determined by HPLC-MS/MS. Results are mean of three donors treated in triplicates ± SEM. The amount of BPDE-N^2-dGuo in the control samples was below the detection limit.

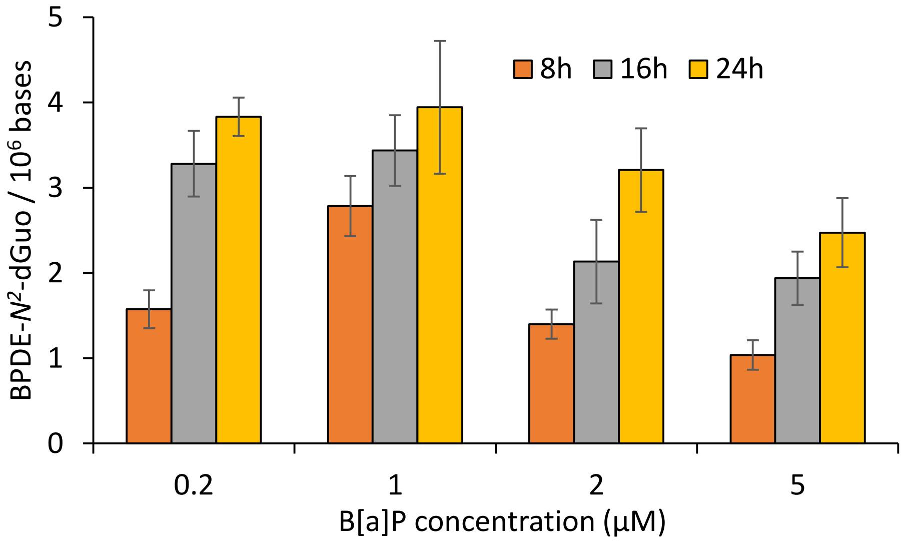

6. Figure S4. HPLC-MS/MS detection of BPDE-N^2-dGuo in skin

The figure below shows typical HPLC-MS/MS chromatograms of the analysis of BPDE-N^2-dGuo in the DNA of skin explants exposed for 48 hours to 100 nmol B[a]P under different conditions. a) standard of BPDE-N^2-dGuo (400 fmol) b) untreated control, c) samples incubated with B[a]P alone, d) samples exposed to 2 MED SSL 1 hour before the beginning of the B[a]P exposure (SSL/B[a]P protocol) and, e) samples exposed to 2 MED 24 hours after the beginning of the B[a]P treatment (B[a]P/SSL protocol). The traces shown are the total ion currents which represent the sum of three signals corresponding to specific fragments produced by BPDE-N^2-dGuo in well-defined ratio. The chromatographic peaks were identified by comparison of the retention time and mass spectrometry features with those of the authentic standard. The amount of analyzed DNA was the similar (< 10% difference) in all samples.

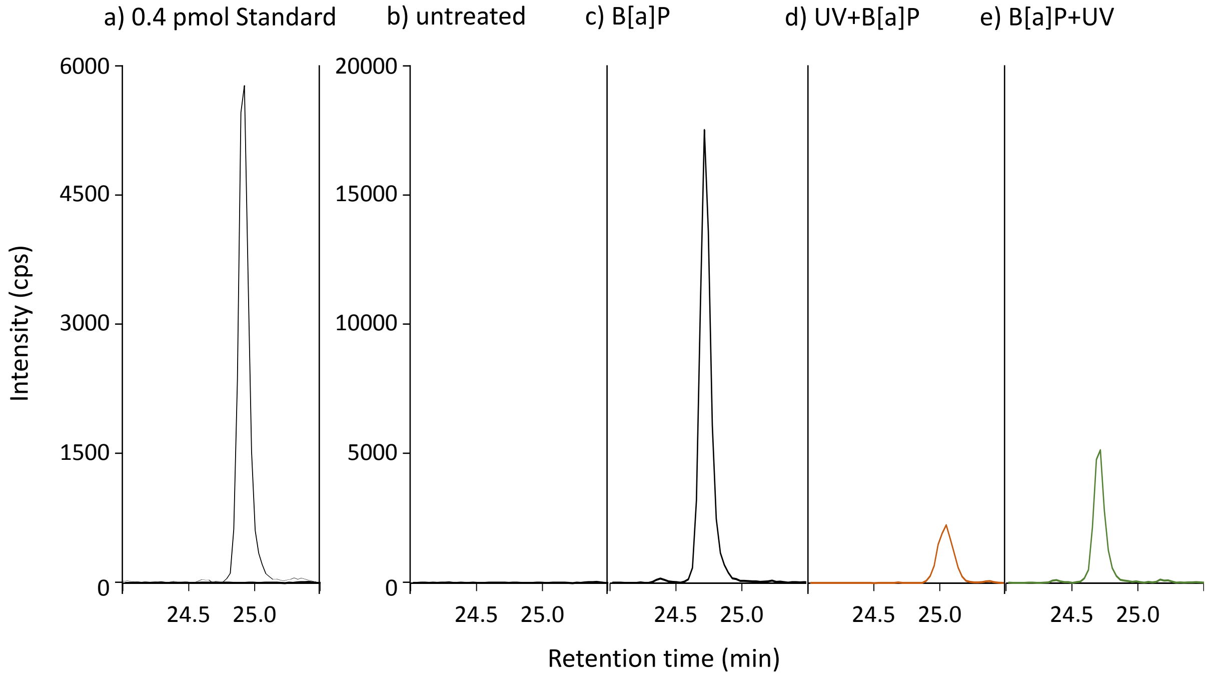

7. Figure S5: Impact of the delay between exposure to SSL and B[a]P treatment on the formation of BPDE-N^2-dGuo in DNA

Skin explants and NHK were treated with the SSL/B[a]P protocol with varying period of times between the end of the irradiation and the addition of B[a]P in fresh medium. Results are compared with the level of adduct in unirradiated samples. In all cases, samples were collected 24 hours after the addition of B[a]P. Values are the mean ± standard deviation (n=6).

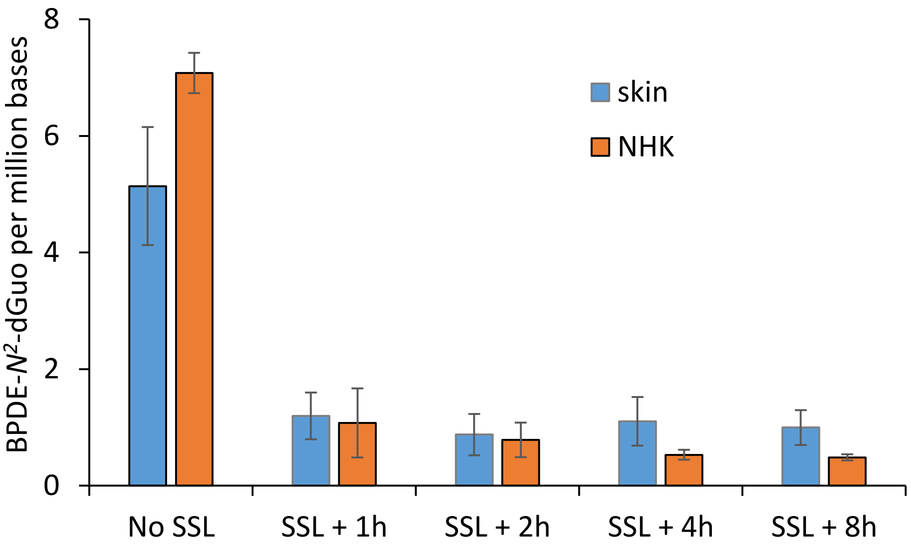

8. Table S3: Toxicity of SSL, UVB and B[a]P in HaCaT cells.

Viability of HaCaT exposed to either SSL, B[a]P, or UVB. Results were obtained via MTT assay performed either 24 or 48 hours after treatment. Reported values are mean ± SEM (n=6).

SSL (MED)	0	1	2	5	10
24h	100±7	93±4	84±2	22±4	17±3
48h	100±8	91±4	95±1	11±2	3±1
BaP (μM)	0	0.5	1	2	5
24h	100±2	100±3	98±5	111±2	102±3
48h	100±5	107±7	100±5	113±4	138±7
UVB (mJ/cm^2)	0	10	25	50	100
24h	100±3	93±2	83±2	76	61±4
48h	100±3	95±3	95±3	98	86±3

9. Figure S6: Formation of BPDE-N^2-dGuo adducts in HaCaT cells co-exposed to UV and B[a]P

HaCaT were exposed in different ways. In the UV/B[a]P protocols, cells were irradiated and B[a]P was added after 1 hour. In the B[a]P/UV protocols, cells were incubated with B[a]P for 24 hours, and then exposed to UV. Control samples only treated with B[a]P were also prepared. The reported values are mean ± standard deviation (n=3).

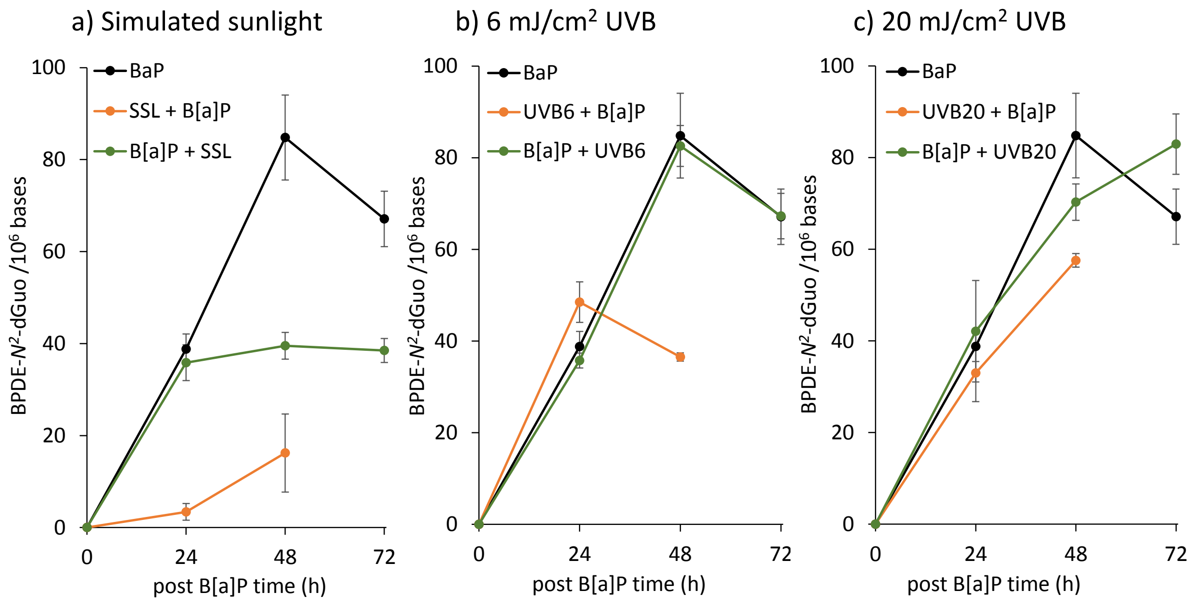

Supplement: Supplementary file 1 — Supplementary Information [file 41598_2018_33031_MOESM1_ESM.pdf]
